# Translation and validation of the medication management patient satisfaction survey: The Lebanese Arabic version

**DOI:** 10.3389/fphar.2023.997103

**Published:** 2023-02-07

**Authors:** Nada Alaa Eddine, James B. Schreiber, Mohamed Ezzat Khamis Amin

**Affiliations:** ^1^ Faculty of Pharmacy, Beirut Arab University, Beirut, Lebanon; ^2^ School of Nursing, Duquesne University, Pittsburgh, PA, United States; ^3^ Faculty of Pharmacy, Alamein International University, Matrouh, Egypt

**Keywords:** patient satisfaction (MeSH), instrument, validation, pharmacy service, Arabic, translation

## Abstract

**Background:** No Arabic translation exists for the medication management patient satisfaction survey (MMPSS), a 10-item psychometrically valid patient satisfaction survey tool developed to assess patient satisfaction for comprehensive medication management. The objective of this study is to translate the medication management patient satisfaction survey into Lebanese Arabic while culturally adapting and assessing the psychometric properties of the translated survey in the outpatient setting.

**Methods:** Guidelines for translation, adaptation, and validation of instruments for cross-cultural healthcare research were followed. The process included forward translation, expert panel review, back-translation, pretesting, and cognitive interviewing. Participants were approached after picking up their medications from the pharmacy at a primary care facility. The medication management patient satisfaction survey was administered verbally by two trained data collectors. Instrument psychometric analyses included testing both for reliability using Cronbach’s alpha (α) and McDonald’s omega (ω) and for construct validity using exploratory factor analysis (EFA). Pearson correlations between items were calculated.

**Results:** During the translation process, the term “clinical pharmacist” was changed to “pharmacist today” for improved understanding. Four items were adapted through minor linguistic modifications. Data were collected from 143 patients. The mean age of participants was 72 years. Participants were mostly females (69%) and had an average of four comorbidities and eight daily medications. Findings from Cronbach’s *α* and McDonald’s ω indicated that the internal consistency among items from one to nine was very strong (*α* = 0.90; ω = 0.90). Exploratory factor analysis indicated that all items are strongly influenced by one factor, except for item six, “My clinical pharmacist is working as a team member with my other healthcare providers” which was the least influenced (loading = 0.44) with the highest uniqueness (0.81). The latent factor captured over 50% of the variance originally observed between variables. Items four and five “My clinical pharmacist helped me find easier ways to take my medicines” and “My clinical pharmacist helped me understand the best ways to take my medicines”, had the strongest correlation (0.77), while the weakest correlation was seen between item six “My clinical pharmacist is working as a team member with my other healthcare providers” and other items.

**Conclusion:** The Lebanese Arabic version of the medication management patient satisfaction survey was produced as a brief tool to serve as a valid and reliable instrument for measuring patient satisfaction with comprehensive medication management services.

## 1 Introduction

Patients’ evaluation of provided care has become a key method of assessing the quality of healthcare services. Patient satisfaction builds on the importance of patient evaluation by assessing whether a patient’s expectations about a health encounter were met (Agency for Healthcare Research and Quality, 2016). It has become a key aspect in assessing the quality and outcomes of patients’ evaluations of healthcare services including those provided by pharmacists. Pharmacists’ cognitive services is a term that has been introduced in 1989 by the cognitive services working group to address the evolution of the pharmacy profession (Working Group Bibliography, 1989). It is defined as “services provided by a pharmacist to or for a patient or health-care professional that are either judgmental or educational in nature rather than technical or informational.” Those services have been expanding in the past decades to include pharmacist-led patient education programs, drug-level monitoring, counseling, and even home visits for patients with chronic conditions (Patwardhan et al., 2014). More recently, the term Comprehensive Medication Management (CMM) has been used to describe “the standard of care that ensures each patient’s medications (whether they are prescription, non-prescription, alternative, traditional, vitamins, or nutritional supplements) are individually assessed to determine that each medication is appropriate for the patient, effective for the medical condition, safe given the comorbidities and other medications being taken, and able to be taken by the patient as intended” (Patient-Centered Primary Care Collaborative, 2012). The provision of CMM entails a care plan that has been tailored for a patient to achieve specifically targeted goals of therapy while following up with patients to determine the achievement of specific patient outcomes. With CMM, the patient has an active role where a patient fully understands, concords with, and participates in the selection of a therapeutic regimen, thus optimizing and individualizing medication use and consequently clinical outcomes.

With this recognition of the patient’s role in CMM, valid and comprehensive measurement of patient satisfaction with pharmacists’ services has become more important than ever. Several instruments have addressed patient satisfaction with pharmacists’ cognitive services with the potential for use in an outpatient setting (Larson and MacKeigan, 1994; Gourley et al., 2001; Larson et al., 2002; Armando et al., 2012; Moon et al., 2016). Many of those instruments assess non-disease specific satisfaction with pharmacists’ services. Others, however, assess satisfaction with pharmacists’ management of a specific disease state such as diabetes (Krass et al., 2009). Quite often those instruments are originally designed in English before being translated into other languages (Yaghoubifard et al., 2016; Shrestha et al., 2020; Fahad et al., 2021). While those instruments are usually easy to administer and score, with little cost, they usually consist of 20–30 items. Validated instruments assessing patient satisfaction in Arabic have usually addressed satisfaction with pharmacy services in the hospital setting (Al-Jumah et al., 2014), or in the community setting but while focusing on “traditional” product-based pharmacy services rather than extended cognitive services (Hasan et al., 2013).

To provide evidence for the contributions of pharmacists to health systems, the Health-systems Alliance for Integrated Medication Management (HAIMM) collaborative aimed to develop and demonstrate measurable quality standards for CMM. To that end, HAIMM members supported the development and validation of a brief 10-item patient satisfaction tool (Moon et al., 2016). The Medication Management Patient Satisfaction Survey (MMPSS), an English patient satisfaction survey tool was developed through a multiphase development process in the United States. Its composition addresses three content areas relating to the pharmacist’s performance in addressing medication related-needs, engaging patient-related outcomes, and overall patient satisfaction. It consists of 10 items (questions), nine of which use a scale from 1 to 4 (strongly agree, agree, disagree, strongly disagree) in addition to a “not applicable option” and asks participants (patients) to evaluate their experiences with the “clinical pharmacist”. The instrument did not provide “Neutral” as an available option, but six items include “not applicable” as a possible response option. The last MMPSS item *“Overall, how would you rate the quality of care and services you received from the clinical pharmacist?”* asks patients to rate their overall quality of care and services on a Likert-scale from 1 to 5 (excellent, very good, good, fair, poor). An additional open-ended item is typically included at the end of the survey that addresses the improvement of services, which is typically not included in quantitative analyses using MMPSS (Moon et al., 2016). At this point, no Arabic version of the MMPSS exists.

With increasing interest in the implementation of medication therapy management pharmacy services in outpatient settings in Arabic-speaking countries including Lebanon (Domiati et al., 2018), it has become a priority to develop necessary tools that can be used properly, accurately, and comprehensively to evaluate those services. There is a growing demand for Lebanese Arabic versions of brief and easy-to-use instruments assessing patient satisfaction with advanced pharmacists’ services such as MMPSS. Thus, this study aimed to translate, culturally adapt and assess the psychometric properties of the MMPSS in the outpatient setting.

## 2 Methods

### 2.1 Research setting and sample inclusion criteria

The study was conducted in a non-governmental charitable association’s primary care facility that is equipped with a pharmacy. The facility is located in Beirut, Lebanon that provides low-charge consultations and free medications to patients of low socioeconomic status. Patients were included in the study immediately after filling their medications if they were 65 years or older, met the World Health Organization’s endorsed definition of polypharmacy where a patient is taking five or more medications (World Health Organization, 2019), picked up their medications themselves, and were cognitively capable of participating in the study. The sample size was calculated based on another research objective for the study that assesses the impact of a pharmacist-led intervention on patient satisfaction.

### 2.2 Ethical consideration

Ethical approval for the study was obtained from the Institutional Review Board (IRB) at Beirut Arab University (protocol number 2022-H-0076-P-M-0465).

### 2.3 Instrument translation

Approval to use a translated version of the Medication Management Satisfaction Survey (MMPSS) in the study was obtained from the author of the original English version (Moon et al., 2016). The MMPSS consists of nine items following a four-point agreement Likert scale, and one item following a five-point Likert scale. The additional open-ended that addresses the improvement of services at the end of the survey was not included in the original MMPSS analysis or the one provided in this paper. The survey was translated using guidelines for the translation, adaptation, and validation of instruments or scales for cross-cultural healthcare research (Sousa et al., 2010) as follows:

#### 2.3.1 Forward translation

The survey was translated from English to Arabic (Lebanese dialect) by the first author who is a Bilingual (Arabic/English) pharmacist and researcher. Second, the third author, another experienced pharmaceutical health services researcher convened with the first author to review the translated version. The two authors agreed that the term “clinical pharmacist” was to be changed to “pharmacist today” for improved understanding by participants.

#### 2.3.2 Expert panel review

Both English and Arabic versions of the survey were sent to four pharmacists including one who is a professor of pharmacotherapy. According to their comments, four items were adapted through minor linguistic modifications. Regarding item four *My clinical pharmacist helped me find easier ways to take my medicines*, one opinion was to clarify this question by adding when and how to take them. This was not adopted, however, to maintain a resemblance with the original version of the survey.

#### 2.3.3 Back-translation

After applying the modifications provided by the expert panel, the Arabic version was sent to an American pharmacist with native proficiency both in English and in Arabic to retranslate it into English. The back-translated survey was compared to the original English one. This comparison indicated a near resemblance between the two versions and, hence, no additional changes were made at this phase.

#### 2.3.4 Pre-testing and cognitive interviewing

The back-translated survey was administered by the first author to a convenience sample of ten patients at the primary care facility including different age groups and genders. The first author read the questions and response options exactly as they appeared in the instrument. Cognitive interviewing was done by both think-aloud (asking participants to verbalize their thoughts as they attempt to answer the instrument items) and verbal probing (asking participants specific questions after the participant answers to seek further information) techniques (Willis, 2004). All items were well understood, and no changes were incorporated. Finally, the instrument was tested on an additional convenience sample of eight patients at the primary care facility using less than five medications. This process resulted in no additional changes to the instrument.

### 2.4 Data collection

Written informed consent was obtained from all participants while explaining to patients that the care they receive at the primary care facility would not be impacted in any way if they choose not to participate. Data collection took place between June and September 2021. Participants were approached after picking up their medications from the pharmacy at the primary care facility. The MMPSS was administered verbally by two trained data collectors. Data collectors were trained to adhere to the agreed-upon introductory script and maintain neutrality while reading survey items in order not to influence participants’ responses. They were also instructed not to answer respondents’ questions regarding the interpretation of survey items to ensure consistency. Role-playing was used in training data collectors to ensure their understanding and adherence to the agreed-upon procedure.

### 2.5 Statistical analyses

#### 2.5.1 Descriptive analysis

Descriptive statistics were used to describe the characteristics of the sample including age, gender, number of comorbidities, and number of medications. Descriptive analyses were done using IBM SPSS 24 ^®^ generating frequencies, as well as means and ranges as relevant.

#### 2.5.2 Instrument psychometric analysis

Instrument psychometric analyses included testing both for reliability and construct validity. Cronbach’s alpha (α) and McDonald’s omega (ω) were used to assess the reliability of the translated instrument from item one to item nine. Pearson correlation between items was calculated and transformed into a correlation matrix.

Construct validity was examined using Exploratory Factor Analysis (EFA). First, Bartlett’s test of sphericity and Kaiser-Meyer-Olkin (KMO) were tested as assumption checks to ensure that factor analysis could be performed. Bartlett’s Test of Sphericity checks the null hypothesis that the correlation matrix is an identity matrix with variables that are not related and, hence, not ideal for EFA. A statistically significant test of P less than 0.05 shows that the correlation matrix for variables studies is not an identity matrix (rejection of the null hypothesis). The KMO tests to see if the partial correlations within generated data are close enough to zero to suggest that there is at least one latent factor underlying the study variables. Ideally, KMO values approach a value of 1.0 while values less than 0.5 are unacceptable. Then, EFA was performed to determine latent factors influencing variables (Schreiber, 2021). Item ten, “*Overall, how would you rate the quality of care and services you received from the pharmacist?”* is different in key aspects from the rest of the instrument. It addresses the general evaluation of the quality of the pharmacist’s services rather than specific aspects of the pharmacist’s service as with other items. It represents an overall composite statement that conceptually and technically measures a different related construct that should be predicted by the construct represented in the first nine items. In light of this, it was excluded from the EFA analysis.

The eigenvalues were computed and arranged in a scree plot in descending order. A scree plot is a graph of eigenvalues against the corresponding number of factors. The number of factors retained is then determined by identifying the point at which the graph shows a sharp drop. The choice of the number of factors to retain was based on parallel analysis, a simulation procedure based on the number of items and sample size that calculates simulated eigenvalues which can be used to compare to eigenvalues from the analysis. There were no missing data on any participant for the 10 items. The hypothesized model was assessed for goodness-of-fit by absolute fit index using Root Mean Squared Error of Approximation (RMSEA) and incremental fit index using Tucker-Lewis Index (TLI) and Bayesian Information Criterion (BIC) (Schreiber, 2017). RMSEA is a measure of the estimated discrepancy between the population and model-implied population covariance matrices per degree of freedom. According to Schreiber (2017) RMSEA values ≤0.05 represent a good fit, values between 0.05 and 0.08 as an adequate fit, and values between 0.08 and 0.10 as a mediocre fit. Values >0.10, however, are not acceptable (Schreiber, 2017). The BIC and TLI approach to model selection favors more parsimonious models over more complex models since it adds a penalty with the increase in the number of parameters that are estimated in the model. TLI ranges between 0 and 1 with recent work tending to show that TLI ranging from 0.90 to 0.95 had the right dimensionality (Clark and Bowles, 2018). BIC is used most often with comparing models but is helpful in the evaluation of smaller models with large values indicating a problem in the model selection. Analyses were done using the online software Jamovi ^®^ with a maximum likelihood extraction in combination with oblimin rotation. The current guidelines from the American Statistical Association concerning *p*-values were followed (Wasserstein et al., 2019). Thus, the *p*-values were provided without using the phrasing “statistically significant.”

## 3 Results

### 3.1 Participants’ characteristics

Of 157 participants approached, 143 patients agreed to participate. The mean age of participants was 72 years. Most of the participants were female (69%). Participants had an average of four comorbidities and were using an average of eight medications daily (Table 1).

**TABLE 1:** Participants’ characteristics and descriptive statistics (N = 143).

		Values
Age	Mean	72
	Standard deviation	5.3
	Range	65–86
Gender	Female	108 (69%)
Body mass index	Mean	29.6
	Standard deviation	4.3
	Range	20–40
Smoking status	Smoker	47 (33%)
Number of comorbidities	Mean	4
	Standard deviation	1.5
	Range	1–9
Number of medications	Mean	8
	Standard deviation	2.2
	Range	5–17

### 3.2 Translation

The translation process was completed within the period from March to June 2021. The final version of the instrument in Lebanese Arabic was the result of all the iterations described in the methods section. Following pre-testing and cognitive interviewing, a final version was produced. All items within that final version were well understood and no changes were considered. The original and Lebanese Arabic versions of MMPSS are provided in Appendix 1.

### 3.3 Instrument psychometric analysis

#### 3.3.1 Reliability analysis

Findings from Cronbach’s *α* and McDonald’s ω indicated that the internal consistency among items from one to nine was very strong (*α* = 0.90; ω = 0.90), (Table 2). A positive correlation was observed between all items in the instrument. Items four and five *“My clinical pharmacist helped me find easier ways to take my medicines”* and *“My clinical pharmacist helped me understand the best ways to take my medicines,”* had the strongest correlation (0.77), while the weakest correlation was seen between item six *“My clinical pharmacist is working as a team member with my other healthcare providers”* and other items (Table 3).

**TABLE 2:** Scale and item reliability statistics (N = 143).

			Cronbach’s *α*	McDonald’s ω
Scale			0.90	0.90
			If item dropped	
	Mean	SD	Cronbach’s α	McDonald’s ω
Item1	2.03	0.64	0.89	0.89
Item2	1.88	0.64	0.88	0.89
Item3	1.90	0.77	0.88	0.89
Item4	1.90	0.64	0.88	0.89
Item5	1.88	0.72	0.88	0.88
Item6	2.17	0.64	0.91	0.91
Item7	2.21	0.66	0.88	0.89
Item8	1.86	0.67	0.89	0.90
Item9	2.33	0.60	0.90	0.90

**TABLE 3:** Correlation matrix for items 1–9 (N = 143).

	Item 1	Item 2	Item 3	Item 4	Item 5	Item 6	Item 7	Item 8	Item 9
Item 1	**1**								
Item 2	**0.61**	**1**							
Item 3	**0.65**	**0.63**	**1**						
Item 4	**0.59**	**0.58**	**0.59**	**1**					
Item 5	**0.58**	**0.67**	**0.71**	**0.77**	**1**				
Item 6	**0.32**	**0.41**	**0.32**	**0.23**	**0.38**	**1**			
Item 7	**0.59**	**0.56**	**0.56**	**0.56**	**0.59**	**0.45**	**1**		
Item 8	**0.44**	**0.5**	**0.54**	**0.48**	**0.55**	**0.22**	**0.55**	**1**	
Item 9	**0.42**	**0.43**	**0.35**	**0.39**	**0.45**	**0.35**	**0.39**	**0.4**	**1**

#### 3.3.2 Appropriateness of conducting an exploratory factor analysis

Bartlett’s test of sphericity and KMO indicated that the variables are related and are ideal for factor analysis (Bartlett’s test *p* < 0.001) and that the degree of information among the variables overlap greatly assuring the presence of a strong partial correlation (KMO 0.90). Hence it was concluded that it was plausible to conduct a factor analysis (Table 4).

**TABLE 4:** KMO measure of sampling adequacy (N = 143).

MSA	Overall	Item1	Item2	Item3	Item4	Item5	Item6	Item7	Item8	Item9
	0.90	0.90	0.94	0.90	0.86	0.85	0.80	0.90	0.91	0.92

#### 3.3.3 Exploratory factor analysis findings

**FIGURE 1:**
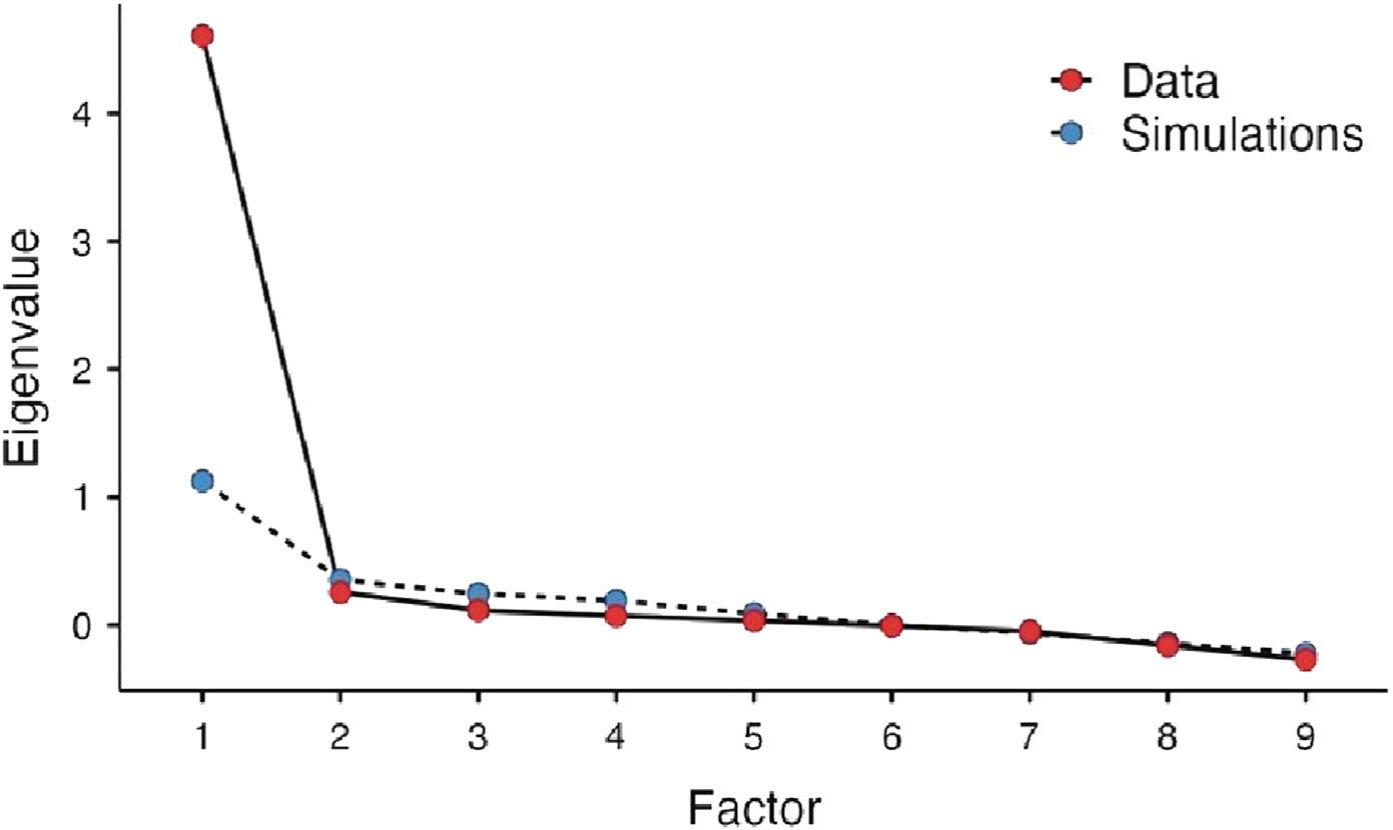
Scree plot of eigenvalues per factor.

EFA indicated that all items are strongly influenced by one factor, except for item six, which was the least influenced (loading = 0.44) with the highest uniqueness (0.81). The latent factor captured over 50% of the variance originally observed between variables (Table 5). A scree plot of the elaborated eigenvalues and parallel analysis with simulated data indicated that only one factor is the best fit for our data (Figure 1). The fact that the difference between the first and second eigenvalue is greater than a factor of three was a further indication for only one factor (Reckase, 1979). As with the original instrument, the revealed factor was labeled “patient satisfaction”. The hypothesized model was assessed as having a marginal fit by absolute fit index (RMSEA: 0.10) and a good fit by incremental fit index (TLI: 0.93). The BIC value for this model is 71.22 (Table 6).

**TABLE 5:** Exploratory factor analysis (N = 143).

	Factor		Uniqueness
	1		
Item1	0.74		0.45
Item2	0.78		0.39
Item3	0.80		0.36
Item4	0.79		0.37
Item5	0.87		0.25
Item6	0.44		0.81
Item7	0.73		0.47
item8	0.65		0.58
Item9	0.53		0.73

Note. “Maximum likelihood” extraction method was used in combination with an “oblimin” rotation.

**TABLE 6:** Model fit measures (N = 143).

RMSEA	RMSEA 90% CI				Model test		
	Lower	Upper	TLI	BIC	χ^2^	Df	P
0.10	0.07	0.13	0.93	−71.22	62.78	27	<.001

## 4 Discussion

With the current expansion and interest in developing pharmacist’s services in Lebanon and other Arab countries, there is a continuing need for comprehensive and easy-to-use patient satisfaction tools that helps in drawing conclusion about the quality of pharmacy services provided by pharmacists. To that end, the Lebanese Arabic version of the MMPSS was successfully translated and cross-culturally adapted in an outpatient setting. The translated instrument demonstrated good psychometric properties (validity and reliability), which were verified using the established methodology. Those psychometric properties were comparable to those reported for the English MMPSS and displayed good acceptability by participants. As with the original MMPS tool, EFA testing of the Lebanese Arabic version resulted in one significant factor labeled “patient satisfaction,” with the scree plot showed a break after the first factor in both versions. In addition, findings from Cronbach’s *α* and McDonald’s ω indicated that as with the original survey, the internal consistency among items from one to nine was very strong. Specifically, Cronbach’s alpha was 0.90 for the Lebanese Arabic version and 0.95 for the original American English version. This is reassuring that the translated MMPSS is equivalent to the original one. Further, the fact that the reliability of the MMPSS continued to be very high after excluding item ten is rather promising as it makes the scale easier to administer for patients, the healthcare team, and researchers.

Further, EFA analysis indicated that item six had a higher uniqueness/lower commonality compared to other MMPSS items. It is possible that the phrasing of this item “*My pharmacist is working as a team member with my other healthcare provider”*, which assesses an interaction of the pharmacist with other health professionals rather than an aspect that is more directly experienced by the patient as with other items, may have contributed to the lower the relevance of this item in the factor model. Despite this uniqueness, the decision to keep the item was made in this analysis because of its conceptual relevance. Still, it would be interesting to see if future translations of the MMPSS would find similar item patterns such as the ones observed here.

An interesting finding in this study is how the word “pharmacist” was understood compared to “clinical pharmacist”. With the original MMPSS, which was tested in the United States, the term “clinical pharmacist” was viewed by focus group patients to better identify CMM pharmacists minimizing confusion with pharmacists who primarily provide medication distribution functions (Moon et al., 2016). The use of the term “clinical pharmacist” in the current study, however, resulted in confusion for participants in the pilot study phase. It is possible that the better comprehension for this term in the original MMPSS was because participants in the United States study had at least one previous exposure to a clinical pharmacist, which improved their familiarity with the term and what it implies.

This Lebanese Arabic translation of the MMPSS resulted in a culturally adapted version that is psychometrically valid and can be used as a pharmacist satisfaction measure, offering an important easy-to-use tool for researchers conducting pharmaceutical health services research. Still, the limitations of this work merit discussion. Lebanese Arabic dialect provides an advantage of improving comprehension with individuals familiar with the language. Researchers and pharmacists who are interested in using this instrument within other Arabic-speaking settings would need to carry out further tool revisions and testing as needed. While the number of participants was adequate for the performed analysis, testing was done in a single institution only. Further validation would confirm the acceptability of this version in other settings of relevance to pharmacists and researchers.

## 5 Conclusion

The Lebanese Arabic version of the MMPSS was produced as a valid and reliable tool to assess patient satisfaction with pharmacist-led CMM. As with the original version, this translation of MMPSS, an easy-to-use and adopt tool for research, can be used in combination with qualitative methods to produce information of key value in evaluating the impact of pharmacists’ services in outpatient settings. Further research can test the utility of the Lebanese Arabic MMPSS in other pharmacy practice settings and along with other measures of quality of cognitive pharmacists’ services.

## Data Availability

The raw data supporting the conclusions of this article will be made available by the authors, without undue reservation.
