# Supplementary material for: Translation and validation of the medication management patient satisfaction survey: The Lebanese Arabic version
Source: Front Pharmacol. 2023 Feb 7;14:997103. doi: 10.3389/fphar.2023.997103 (PMC9941315; doi:10.3389/fphar.2023.997103)
Supplement: Supplementary file 1 [file Table1.DOCX]

Supplementary Material

**Appendix 1**

**Medication Management Patient Satisfaction Survey in English**

1. My clinical pharmacist helped me to understand why I am taking each of my medicines.

🞎 Strongly agree

🞎 Agree

🞎 Disagree

🞎 Strongly disagree

🞎 Not applicable

2. My clinical pharmacist helped me understand how to know if my medicines are working.

🞎 Strongly agree

🞎 Agree

🞎 Disagree

🞎 Strongly disagree

🞎 Not applicable

3. My clinical pharmacist made certain that my medicines are safe (knowing possible side effects of my medicines and avoiding drug interactions).

🞎 Strongly agree

🞎 Agree

🞎 Disagree

🞎 Strongly disagree

🞎 Not applicable

4. My clinical pharmacist helped me find easier ways to take my medicines.

🞎 Strongly agree

🞎 Agree

🞎 Disagree

🞎 Strongly disagree

🞎 Not applicable

5. My clinical pharmacist helped me understand the best ways to take my medicines.

🞎 Strongly agree

🞎 Agree

🞎 Disagree

🞎 Strongly disagree

🞎 Not applicable

6. My clinical pharmacist is working as a team member with my other health care providers.

🞎 Strongly agree

🞎 Agree

🞎 Disagree

🞎 Strongly disagree

7. After talking with my clinical pharmacist, I feel more confident to manage my medicines.

🞎 Strongly agree

🞎 Agree

🞎 Disagree

🞎 Strongly disagree

8. My clinical pharmacist listened to concerns about my medicines.

🞎 Strongly agree

🞎 Agree

🞎 Disagree

🞎 Strongly disagree

🞎 Not applicable

9. I would recommend my clinical pharmacist to a family member or friend.

🞎 Strongly agree

🞎 Agree

🞎 Disagree

🞎 Strongly disagree

10. Overall, how would you rate the quality of care and services you received from the clinical pharmacist?

🞎 Excellent

🞎 Very good

🞎 Good

🞎 Fair

🞎 Poor

Moon, J., Kolar, C., Brummel, A., Ekstrand, M., Holtan, H., and Rehrauer, D. (2016). Development and validation of a patient satisfaction survey for comprehensive medication management. J Manag. Care. Spec. Pharm. 22, 81–86. doi: 10.18553/jmcp.2016.22.1.81.

Lebanese Arabic version of Medication Management Patient Satisfaction Survey

استبيان رضا المرضى عن إدارة الأدوية

1-

ساعدني الصيدلي اليوم إفهم ليه عم باخد كل دوا من أدويتي:

ما اتطبق ما بوافق ابدا ما بوافق بوافق كتير بوافق

2-

ساعدني الصيدلي اليوم إفهم كيف فيي أعرف إذا ادويتي عم تفيدني (هي فعالة):

ما اتطبق ما بوافق ابدا ما بوافق بوافق كتير بوافق

3-

تأكد الصيدلي اليوم انو ادويتي آمنة (أعرف اثارها الجانبية الممكنة وإتفادى تفاعلها مع بعضا):

ما اتطبق ما بوافق ابدا ما بوافق بوافق كتير بوافق

4-

ساعدني الصيدلي اليوم لاقي طرق أسهل لأخد ادويتي:

ما اتطبق ما بوافق ابدا ما بوافق بوافق كتير بوافق

5-

ساعدني الصيدلي اليوم افهم أحسن الطرق لأخد ادويتي:

ما اتطبق ما بوافق ابدا ما بوافق بوافق كتير بوافق

6-

عم يشتغل الصيدلي كعضو في فريق مع الطاقم الطبي الخاص فيي:

ما بوافق ابدا ما بوافق بوافق كتير بوافق

7-

بعد ما حكيت مع الصيدلي اليوم صار عندي ثقة أكتر كيف فيي نظم أدويتي بطريقة أحسن:

ما بوافق ابدا ما بوافق بوافق كتير بوافق

8-

استمع الصيدلي اليوم للمخاوف اللي عندي ياها تجاه أدويتي:

ما اتطبق ما بوافق ابدا ما بوافق بوافق كتير بوافق

9-

بنصح الأهل والأصحاب بالصيدلي اللي تعاملت معو اليوم:

ما بوافق ابدا ما بوافق بوافق كتير بوافق

10-

بشكل عام كيف بتقيم جودة الرعاية والخدمة اللي قدملك اياها الصيدلي اليوم؟

ما منيحةمقبولة منيحة كتير منيحة ممتازة
